# The General Hospital, Toronto

**Published:** 1910-04-02

**Authors:** 


### SPECIAL ARTICLE.

### THE GENERAL HOSPITAL, TORONTO.

FROM AN ENGLISH CORRESPONDENT.

### I.—THE OLD BUILDINGS.

Toronto may be termed the metropolis of the English speaking part of Canada. She is the second in size and the youngest in point of age, but Quebec, the oldest, is wholly French, and Montreal is as much, if not more, French than English. Toronto has increased with remarkable rapidity, and is still increasing. Her population is 330,000 or thereabouts. According to Dr. James F. W. Ross, Toronto's first general hospital was established in 1819 through the efforts of the military surgeons, when the population of the place, then known as York, was about a thousand. The Chairman of the Medical Board was one of the best known physicians who has ever lived in Toronto—Dr. Christopher Widmer—and he held the position for thirty-five years. Dr. Grant Powell superintended the erection of the first hospital. By his direction a spacious plain two-story red brick building was erected; the main building was 107 feet long and 66 feet wide. It was endowed with land within the city limits, and had also a grant from the Legislature. In 1850 there were about a hundred patients in the institution.

Soon after this date the second and present general hospital was built. It occupies a space of four acres on the north side of Gerrard Street East, perhaps a mile distant from the centre of the city. The hospital buildings are constructed in rectangular form, and are 170 feet by 120 feet. The main building is of white brick with stone dressing, three storied, with a mansard roof, a central tower 100 feet in height, and two smaller towers at each angle of the front elevation. Externally the building is certainly one of the most picturesque hospitals of the world. Covered with ivy and Virginia creeper, and standing back from the roadway, amidst trees and greenery, it presents an eminently comfortable and home-like appearance. Connected with the main building by bridges on either side are the fever hospital and the Mercer Eye and Ear Infirmary. In the north-west angle of the grounds is the Burnside Lying-in Hospital, which is supported by voluntary contributions and a yearly Government grant. The building of the Burnside Hospital was erected by money left by a quack who died intestate, and whose estate was expended by the Government in this manner. This building, as well as the Eye and Ear Infirmary and Fever Hospital, is of the same style and material as the main structure. It should also be mentioned that, situated between the main building and the Burnside Hospital, is the gynæcological department, with beds and private ward accommodation for 38 women.

The interior of the hospital itself does not belie its outward appearance. It is a rambling, almost cosy, old building. The first floor in front is largely taken up with administrative offices; for its mode of administration, although different from that of English hospitals, entails perhaps even more work upon the staff. Before proceeding further with a description of the building, it may be as well to explain broadly the manner in which the hospital is administered, and the sources from which it draws its revenue. In the first instance, certain lands were granted by the Crown vested in trustees. In 1847 these trustees were incorporated by Act of Parliament. At the present time the Trust holds the residue of the original hospital grant, together with property since given by friends of the hospital. But the income derived from the Trust constitues only a comparatively small part of the hospital's yearly revenue.

The Canadians, although sufficiently patriotic, follow in many respects the lead of the United States rather than that of the Mother Country, and especially in the case of hospital management. Free indoor treatment is almost unknown in the hospitals of the American Continent. Toronto General Hospital has a capacity of 400 beds, and of these, when your correspondent paid a visit to the institution recently, exactly seven were occupied by persons who paid nothing for treatment. The 340 occupied beds were thus tabulated. Private patients, 32 beds at charges varying from $16 (£3 6s.) to $31 (£6 10s.) per week, 17 beds occupied. Semi-private patients, 45 beds, at charges varying from $12.50 (£2 10s.) to $13.50 (£2 15s.) per week, 28 beds occupied. Semi-public patients, 30 beds, at $7 (£1 10s.) per bed per week, 22 occupied. Public ward, 138 beds occupied, at $4.90 (£1) per bed a week. Municipal order, 128 beds occupied, at $4.90 (£1) per bed a week. In the public ward and the municipal ward together the bed capacity is 298, of which 266 were occupied. In the public wards the patients pay for themselves or are paid for by relations or friends. In the municipal order wards the patients are paid for by the City of Toronto. The city gives orders to charitable agencies and philanthropists, who grant them to deserving persons in need of medical or surgical aid and unable to afford it for themselves. It will thus be observed that with the exception of seven people the hospital on October 30 last was receiving payment for all its patients at the rate of about £440 per week. This sum will go a very considerable way towards defraying expenses. It should also be mentioned that the Government of Ontario contributes towards the keep of each patient in the public and municipal order wards the sum of ·20 cents (tenpence) a day, which will bring up the rate of payment the hospital of Toronto was receiving for its patients on October 30 to £517 per week. One dollar per week is charged in addition to the ordinary rate of payment for each surgical case. Outside medical practitioners are allowed to visit patients in private and semi-private wards, but are not allowed access to other parts of hospital. The main difference then in the management of American hospitals and of the hospitals of Great Britain is that the former are self-supporting to a very large extent. The view is held in America that the system is the best for all concerned, that it fosters independence and self-respect in the patients, that the private wards provide the best possible treatment under the most favourable conditions to a class of patients who have not the proper facilities for, nor can they afford the expense of, being treated at home. It is argued that the system in vogue in the hospitals of Great Britain tends to pauperise the people and to lower their sense of dignity. Furthermore, that there are few, if any, suitable places in Great Britain in which persons of moderate means can receive the best medical or surgical aid under good conditions.

The hospital is administered by a trustee board appointed severally by the subscribers, by the Government, by the University, and by the City Council. There are also a number of honorary or visiting governors, each of whom contributes $100 yearly. There is a somewhat exceptionally large staff of consulting and active physicians and surgeons. The direct management of the hospital is in the hands, as is generally the case in America, of a medical superintendent.

The main hospital building contains nineteen large public wards of varying size, and about twenty private wards. The public wards are roomy, airy, and appear, from a somewhat cursory inspection to be well ventilated. A hospital, however, which is nearer sixty than fifty years old cannot be expected to contain all the modern improvements. It would be easy to point out numerous defects in construction viewed from the up-to-date medical and sanitary standpoint. For instance, some of the wards have pillars supporting the ceiling running down the length of the rooms, and other faults of detail which would be looked upon with a censorious eye by the enthusiastic hospital reformer. The private wards are comfortable, but are open to similar criticism. The greatest drawback to the institution is that it is very much overcrowded. At the present time use is being made of attics in the roof for the accommodation of patients. It is true that these are airy and that the patients do well in them. Indeed, the results from the hospital as a whole are exceedingly good; but at the same time it is hardly fitting that a city of the size and importance of Toronto, and, moreover, one which contains a large number of medical students, should not have a hospital building in keeping with its manifest needs. Again, there is generally a considerable amount of typhoid fever in Toronto, and in the general hospital these patients are intermingled promiscuously with other medical cases.

The operating theatre is in the centre of the building, flanked on either side by a wing. It is a thoroughly modern theatre, properly equipped, lighted, ventilated, and furnished. There is a pathological department with fairly good laboratories. Also there is an immunisation department, in which Sir Almroth Wright's theories and teaching are carried into effect by one of his most distinguished Canadian disciples, it is stated, with most satisfactory results. A ward for nervous diseases, for those suffering from various forms of nervous affections, such as neurasthenia, hysteria, obsessions, delusions, etc., but who are not really insane, was established in the Toronto General Hospital some one or two years ago. This departure is again one introduced from the United States, where psychiatric wards for the observation of those who may be on the borderland of insanity have been established in the hospitals of all the large cities during the past five or six years. One of the most valuable innovations which has been carried out in the Toronto Hospital is the establishment of a tuberculosis clinic. The work performed under the auspices of this clinic has been most satisfactory. Alcoholics are admitted for treatment into the hospital in rather large numbers; but no ward is specially set apart for their accommodation. The Mercer Eye and Ear Infirmary is a fairly good building, with from fifteen to twenty beds. The gynæcological pavilion is a well built and well arranged annexe, with accommodation for about thirty-seven women, including nine private wards. During the year 1908 there were a large number of ectopic pregnancies. The Burnside Lying-in Home contains five private wards, semi-private wards, and public ward. During the year 1908, 297 patients were admitted, of whom 257 were cured and three died. There was a complete absence of septicæmia. In connection with the hospital there is a large and excellent training school for nurses. In 1908 the staff of the school consisted of two assistants, one night supervisor, six graduate head nurses, 104 pupil nurses, and eight probationers. The number of patients admitted during the year 1908 was 4,005, number of patients remaining from 1907 was 322, number of infants born in 1908 was 263, making a total of

Taking into consideration the age of the hospital, the defects of construction, and its lack of accommodation, the results recorded during the many years of existence have been wonderfully good. Its administration at the present time is excellent in every respect; but those responsible for its control cannot make bricks without straw, and it has been painfully obvious for some time that affairs cannot go on under existing conditions much longer.

### II.—THE PROJECTED NEW BUILDINGS.

The educational facilities as a teaching school provided by the hospital are lamentably behind the times. The number of students walking the hospital is about 325. In short, it has been decided that it is of no use to continue to patch up the old buildings, except for immediate requirements, but that suitable buildings must be provided for the modern and scientific treatment of the sick, and to afford ample opportunities for the educational work of the now united schools of medicine in the University of Toronto.

Your correspondent a few days ago discussed the matter with Mr. J. W. Flavelle, LL.D., the chairman of the Hospital Board, with Mr. P. C. Larkin, vice-chairman, and with Dr. J. U. E. Brown, superintendent, and was informed that in consequence of the ready response to appeals for assistance the decision was quickly made to erect a new hospital. Matters have now advanced so far that a site has been purchased on College Street, near the Children's Hospital, and almost opposite to the buildings of the University of Toronto. The area of the ground is about nine acres. The ground is being rapidly cleared and plans have been drawn for the erection of the hospital. The projected buildings will be complete in every detail. According to a bird's-eye view of the present plans the main building will be three stories in height and will have a frontage on College Street of more than 700 feet. The administrative building will be in the centre, and on one side will be the part devoted to surgical cases, and on the other side that given up to medical cases. At the back on one side will be a large building for use as a nurses' home, and on the other side a still larger building for the use of the students, which will contain laboratories, lecture rooms, and the usual equipment of such places.

There will be buildings for the gynæcological department for the nose and throat department; in fact, the buildings will be suitable for the work of a large city hospital and teaching school in every conceivable respect. Accommodation for 500 patients or thereabouts will be provided in the buildings. One point was particularly impressed upon the writer that, although the buildings will be well built, fireproof, and sightly, no money whatever will be wasted on needless ornamentation, either on the outside or inside. It is anticipated that the new hospital buildings will be completed in three years' time and that they will cost with site more than £400,000. It should be said that in the planning of the new buildings special attention has been paid to the requirements of private patients. A handsome pavilion will be erected at the back of the main building for the accommodation of those who can afford to pay well for medical or surgical treatment. A feature of the proposed hospital which must forcibly strike one who is accustomed to the majority of the hospitals in the big cities of the old country is the spaciousness of the site. The road which the hospital will face is a beautiful residential road, with shady trees on each side. The hospital will stand back some little distance from the road, and behind will have quite a good-sized expanse of grass and trees, with buildings dotted here and there. The roofs of most of the buildings will be flat, and will be utilised in the summer time as roof gardens for the benefit of the patients. The situation is ideal. It is near the city, and it is near the University with its three or four hundred medical students. It may be said that the out-patients' department is large, about ten thousand out-patients having been treated at the present hospital in 1908. The out-patients' department in the new hospital will be ample and well arranged.

Reference has been made more than once to the excellent manner in which the internal economy, so to speak, of the hospital is controlled. This is no doubt chiefly due to the high administrative abilities of Dr. J. U. E. Brown, the superintendent. He has, among other methods for curtailing expenses, devised, or rather improved upon, a means for checking accounts which renders carelessness on the part of nurses, on the part of tradesmen, or on the part of any of those concerned in the handling of provisions or stores impossible. At least, if mistakes or laxity occur, they are certain to be detected, and thus are unlikely to occur again. The cost per caput of patients in the Toronto General Hospital is under $1.50 (6s.) per day, which compares very favourably with the cost in the majority of American hospitals.

It must not be forgotten that the present general hospital of Toronto has done splendid work and has turned out many good physicians and surgeons. The truth is that it has outlived its usefulness. There can be no doubt that Toronto as a medical school has a magnificent future, but to train properly the students a modern, well-equipped hospital is necessary, as it is also necessary to the rapidly increasing population.

